# Diseases of the Heart and Circulation

**Published:** 1903-01-17

**Authors:** 


### DISEASES OF THE HEART AND CIRCULATION.

(*Concluded from page* 251.)

**Dilatation of the Heart in Rheumatism.**—Fisher,[4] in a review of the effects of the heart in rheumatism, says that dilatation of the heart, ending in death, may occasionally occur without valvular disease or general adhesion of the pericardium being present; and in rare instances hypertrophy of the heart may occur without valvular disease or general adhesion of the pericardium. The causation of dilatation and of hypertrophy of the heart associated with pericarditis or with general adhesion of the pericardium is probably to be found in poisoning of the cardiac muscle. Slight weakening of the myocardium may be shown by loss of physical energy, by attacks of cardiac pain, or by tachycardia. Thus a child aged 4 years complained of pain over the heart, and often turned pale. Physical examination of the heart revealed nothing noteworthy, but it was beating rapidly, 148 per minute. There was a history of pain and swelling of the joints, and another attack of arthritis occurred while she was under treatment.

**Mitral Stenosis.**—Bryant[5] shows that pulmonary incompetence and dilatation and atheroma of the pulmonary arteries may result from mitral stenosis. If this becomes extreme, the blood pressure is raised in the pulmonary circuit to such an extent that the pulmonary arteries may become dilated, hypertrophied, and athromatous. The pulmonary orifice may subsequently become incompetent, so that regurgitation occurs during diastole. This produces an early diastolic murmur, best heard in the third left intercostal space midway between the left border of the sternum and the left nipple line. Sometimes, though this evidence has been obvious during life, there is no post-mortem evidence of regurgitation; this is due to the fact that as soon as the tension is relieved (by death) the artery, whose elasticity has not been permanently injured, contracts to its normal size. Mitral stenosis is not the only cause of pulmonary regurgitation, for Bond and Trevor[6] have recorded a case due to chronic bronchitis and emphysema. The murmur was a diastolic one in the third, fourth, and fifth spaces to the left of the sternum, not audible immediately along the border of the sternum, but a little external to this. They remark that such a murmur may be erroneously thought due to aortic disease, or correctly diagnosed but the cause mistaken.

**Malignant Endocarditis.**—Poynton and Beale[7] have recently brought forward evidence that there is a group of cases of malignant endocarditis which is rheumatic in nature. They state that clinical and pathological experience point to this probability, which is confirmed by the following bacteriological evidence. Ordinary rheumatic endocarditis and arthritis are, as they have already pointed out, due to a diplococcus. Now, from some cases of malignant endocarditis a diplococcus can be isolated, which is indistinguishable from the rheumatic one; and injection of cultures into rabbits produces not only malignant endocarditis but a condition which is clinically rheumatic fever. Further, the diplococcus rheumaticus when passed through a series of rabbits will produce malignant endocarditis, which cannot be distinguished from that produced by inoculation of the diplococcus of some cases of malignant endocarditis. Thus every grade of valvulitis, from simple to malignant, may be produced by the diplococcus rheumaticus.

**Digitalis in Heart Disease.**—According to Allan Jones[8] it is of great importance to give nitroglycerine in combination with digitalis. For instance, a patient was liable to arginoid attacks, as a result of coronary degeneration. Digitalis, in ♏10 doses was given every four hours with no improvement in the condition, and finally the heart became very slow and almost stopped. Nitroglycerine was now added to the medicine, and then the annoying symptoms disappeared. Another important accessory to digitalis, particularly in old people, is the use of iodides. Balfour[9] gives small doses of digitalis for long periods in cases of senile heart to tone up and strengthen the cardiac muscle by improving its nutrition. The process is a slow one, and the benefit is not for a time very obvious to the patient, but if he is willing to persist in taking the medicine great improvement becomes manifest.

**Congenital Stenosis of the Aorta at the Isthmus** is a rare congenital abnormality, of which there are but few cases on record. Libman[10] has recorded a case in which life was prolonged to 63 years. The average duration, however, is only 34 years. The stenosis takes place at the opening of the ductus arteriosus, and may be partial or complete. The collateral circulation is carried on mainly by branches of the subclavian arteries, communicating with the intercostal arteries and with some of the branches of the external iliacs. The development of large anastomosing arteries under the skin of the chest and abdomen is the result. The diagnostic signs are: hypertrophy of the left ventricle; a systolic murmur heard over the heart; a disparity between the pulsation in the upper and lower extremities, and the presence of pulsating vessels under the skin of the chest and abdomen in which the wave is from above downwards.

**Heart in Pregnancy.**—Stengel and Stanton[11] state that on delivery there is a movement of the apex of the heart downward and inward. In cases where there is a large and tympanitic abdomen this downward movement is not so marked. During pregnancy there is an enlargement of the heart upward and to the left, probably due to an enlargement of the conus arteriosus. The displacement of the apex of the heart is thus not due to hypertrophy of the left ventricle, but to displacement caused by the upward movement of the diaphragm from pressure, and the increase in size takes place only in the right side of the heart and the conus arteriosus.

**Death in Unruptured Aneurysms.** — Arnold[12] reviews the causes of death in cases of thoracic aneurysms which do not rupture—an important subject, insomuch as only about half the cases end in rupture. Such unruptured aneurysms may be divided into three classes: 1. Those in which it is not the important factor in the fatal result, where the patients die from some intercurrent disease whose origin cannot properly be ascribed to the aneurysm. 2. Cases in which death ensues from disease of the circulatory system not caused by direct pressure of the aneurysmal tumour. Thus, patients may die from arteriosclerosis, by the plugging of the arteries from thrombosis or embolism. The source of an embolus may be the aneurysm itself, or from the heart which has undergone dilatation owing to the aneurysm. 3. Cases in which death is due, directly or indirectly, to pressure of the aneurysmal tumour upon organs of vital importance, includes by far the majority of cases. Thus pressure on peripheral nerves may cause pain, or cough, or dyspnœa, which may help to wear the patient's life away, or it may cause laryngeal spasm or paralysis. Pressure on the bronchial arteries may cause gangrene of the lung. The trachea or bronchi are frequently pressed upon, so that death is caused by suffocation or indirectly from secondary changes in the lung caused by retention of secretions and secondary purulent bronchitis. Œsophageal obstruction may lead to death from starvation.

**Venesection.**—Favill[13] records some interesting cases where venesection probably saved the patient's life. Thus, an elderly man caught a severe cold. Six hours after the first symptoms showed themselves râles could be heard all over the chest, whilst the heart sounds were almost inaudible, and the pulse was exceedingly feeble. The symptoms were relieved by blood-letting, and once the heart passed the preliminary stage of inadequacy it reacted very well, and all the unfavourable conditions passed off. Similarly good effects could be obtained in pneumonia, during the period of congestion, just before hepatisation sets in, when the right side of the heart is seriously embarrassed.

[4] Lancet, June 7. [5] Practitioner, July. [6] Lancet, Oct. 4. [7] Practitioner, July. [8] Jour. Med. Assoc. [9] Quart. Med. Jour., May. [10] Med. Rec., May 31. [11] Med. Rec., May 10. [12] Med. Chron., June. [13] Jour. Amer. Assoc., June 28.

